# Supplementary material for: Microbiological and clinical characteristics of hypervirulent Klebsiella pneumoniae isolated from patients in tertiary centers: a retrospective study
Source: PeerJ. 2025 Oct 16;13:e20198. doi: 10.7717/peerj.20198 (PMC12535742; doi:10.7717/peerj.20198)
Supplement: Supplemental Information 1 [file peerj-13-20198-s001.docx]

| Target gene | Sequence (5’-3’) | | Size (bp) | Reference |
| --- | --- | --- | --- | --- |
| *rmpA* | Forward | ACTGGGCTACCTCTGCTTCA | 535 | (Liu and Guo, 2019) |
|  | Reverse | CTTGCATGAGCCATCTTTCA |  |  |
| *rmpA2* | Forward | CTTTATGTGCAATAAG-GATGTT | 452 | (Liu and Guo, 2019) |
|  | Reverse | CCTCCTGGAGAGTAAGCATT |  |  |
| *iucA* | Forward | AATCAATGGCTATTCCCGCTG | 239 | (Sanikhani et al., 2021) |
|  | Reverse | CGCTTCACTTCTTTCACTGACAGG |  |  |
| *peg-344* | Forward | CTTGAAACTATCCCTCCAGTC | 508 | (Russo et al., 2018) |
|  | Reverse | CCAGCGAAAGAATAACCCC |  |  |
| *magA* | Forward | GGTGCTCTTTACATCATTGC | 1282 | (Liu and Guo, 2019) |
|  | Reverse | GCAATGGCCATTTGCGTTAG |  |  |
| K1 | Forward | GTAGGTATTGCAAGCCATGC | 1048 | (Liu and Guo, 2019) |
|  | Reverse | GCCCAGGTTAATGAATCCGT |  |  |
| K2 | Forward | GGAGCCATTTGAATTCGGTG | 1112 | (Liu and Guo, 2019) |
|  | Reverse | TCCCTAGCACTGGCTTAAGT |  |  |
| *gapA* | Forward | TGAAATATG ACTCCACTCACGG | 636 | (Diancourt et al., 2005) |
|  | Reverse | CTTCAGAAGCGGCTTTGATGGCTT |  |  |

**Table 1. Primers used for the optimization of multiplex PCR assay**
