# Supplementary figures and images for: Microbiological and clinical characteristics of hypervirulent Klebsiella pneumoniae isolated from patients in tertiary centers: a retrospective study
Source: PeerJ. 2025 Oct 16;13:e20198. doi: 10.7717/peerj.20198 (PMC12535742; doi:10.7717/peerj.20198)

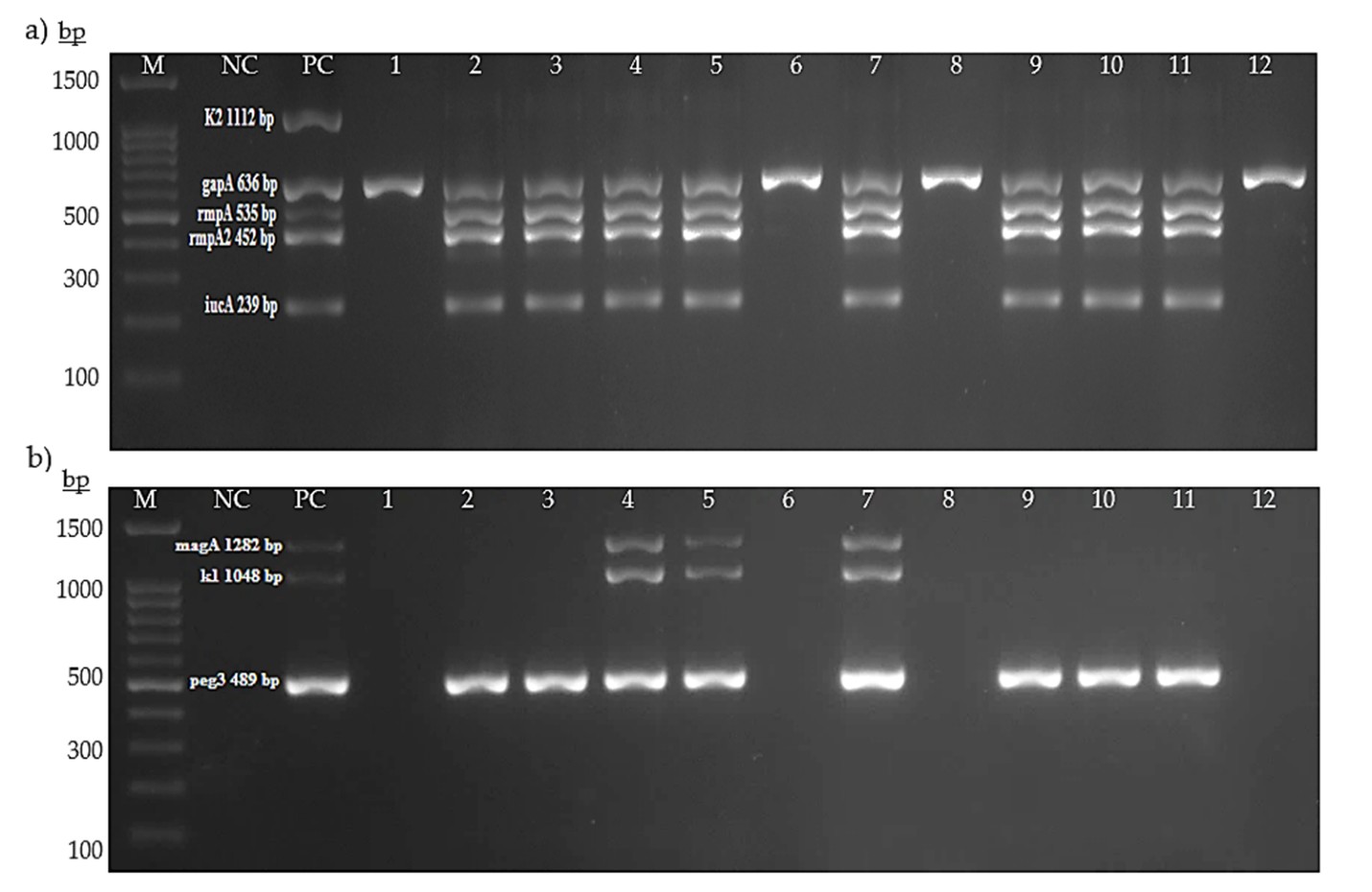

Supplement: Supplemental Information 2 — Assay 1 and Assay 2 targeted K2 serotype (1112 bp), gapA (636 bp), rmpA (535 bp), rmpA2 (452 bp) and iucA (239 bp); and K1 serotype (1048 bp), magA (1282 bp), and peg-344 (636 bp), respectively. Lane M: 1000plus bp DNA ladder, lane NC: negative control, lane PC: positive control, lanes 1312: K. pneumoniae isolates. [file peerj-13-20198-s002.jpg]
