# Supplementary material for: Microbiological and clinical characteristics of hypervirulent Klebsiella pneumoniae isolated from patients in tertiary centers: a retrospective study
Source: PeerJ. 2025 Oct 16;13:e20198. doi: 10.7717/peerj.20198 (PMC12535742; doi:10.7717/peerj.20198)
Supplement: Supplemental Information 4 [file peerj-13-20198-s004.doc]

STROBE Statement—Checklist of items that should be included in reports of ***cross-sectional studies***

|  | Item No | Recommendation | Page number | Remark |
| --- | --- | --- | --- | --- |
| **Title and abstract** | 1 | (*a*) Indicate the study’s design with a commonly used term in the title or the abstract | 1 | Cross-sectional, retrospective study as stated in the Abstract section. |
|  |  | (*b*) Provide in the abstract an informative and balanced summary of what was done and what was found | 1 | Included in Abstract section. |
| Introduction | | |  |  |
| Background/rationale | 2 | Explain the scientific background and rationale for the investigation being reported | 2 | Included in the Introduction section. |
| Objectives | 3 | State specific objectives, including any prespecified hypotheses | 2 | Included in the Introduction section. |
| Methods | | |  |  |
| Study design | 4 | Present key elements of study design early in the paper | 3 | Included in Study design section. |
| Setting | 5 | Describe the setting, locations, and relevant dates, including periods of recruitment, exposure, follow-up, and data collection | 3 | Included in Study design section. |
| Participants | 6 | (*a*) Give the eligibility criteria, and the sources and methods of selection of participants | 3 | Included in Study design section. |
| Variables | 7 | Clearly define all outcomes, exposures, predictors, potential confounders, and effect modifiers. Give diagnostic criteria, if applicable | 3 | Included in Study design section. |
| Data sources/ measurement | 8* | For each variable of interest, give sources of data and details of methods of assessment (measurement). Describe comparability of assessment methods if there is more than one group | 3 | Included in Study design section. Comparability of assessment methods is not applicable. |
| Bias | 9 | Describe any efforts to address potential sources of bias | 3 | Included in Study design section. |
| Study size | 10 | Explain how the study size was arrived at | 3 | Included in Study design section. |
| Quantitative variables | 11 | Explain how quantitative variables were handled in the analyses. If applicable, describe which groupings were chosen and why | 4 | Included in Statistical analysis section. |
| Statistical methods | 12 | (*a*) Describe all statistical methods, including those used to control for confounding | 4 | Included in Statistical analysis section. |
|  |  | (*b*) Describe any methods used to examine subgroups and interactions | 4 | Included in Statistical analysis section. |
|  |  | (*c*) Explain how missing data were addressed |  | Not applicable. |
|  |  | (*d*) If applicable, describe analytical methods taking account of sampling strategy |  |  |
|  |  | (*e*) Describe any sensitivity analyses |  | Not applicable. |
| Results | | |  |  |
| Participants | 13* | (a) Report numbers of individuals at each stage of study—eg numbers potentially eligible, examined for eligibility, confirmed eligible, included in the study, completing follow-up, and analysed | 4 | Included in Social-demographic data and clinical presentations of patients infected with hvKp section. |
|  |  | (b) Give reasons for non-participation at each stage |  | Not applicable. |
|  |  | (c) Consider use of a flow diagram |  | Not applicable. |
| Descriptive data | 14* | (a) Give characteristics of study participants (eg demographic, clinical, social) and information on exposures and potential confounders | 4 | Included in Social-demographic data and clinical presentations of patients infected with hvKp section. |
|  |  | (b) Indicate number of participants with missing data for each variable of interest |  | Not applicable. |
| Outcome data | 15* | Report numbers of outcome events or summary measures | 4 and 5 | Included in all sections under results. |
| Main results | 16 | (*a*) Give unadjusted estimates and, if applicable, confounder-adjusted estimates and their precision (eg, 95% confidence interval). Make clear which confounders were adjusted for and why they were included | 5 | Included in Distribution of virulence genes associated with K1 and K2 capsular serotypes. |
|  |  | (*b*) Report category boundaries when continuous variables were categorized | 4 and 5 | Included in all sections under results. |
|  |  | (*c*) If relevant, consider translating estimates of relative risk into absolute risk for a meaningful time period |  | Not applicable. |
| Other analyses | 17 | Report other analyses done—eg analyses of subgroups and interactions, and sensitivity analyses | 5 | Included in Distribution of virulence genes associated with hypermucoviscous *K. pneumoniae* (hmKp) and non-hypermucoviscous *K. pneumoniae* (non-hmKp) and Distribution of virulence genes associated with K1 and K2 capsular serotypes section. Sensitivity analyses not applicable. |
| Discussion | | |  |  |
| Key results | 18 | Summarise key results with reference to study objectives | 6 and 7 | Included in Discussion section. |
| Limitations | 19 | Discuss limitations of the study, taking into account sources of potential bias or imprecision. Discuss both direction and magnitude of any potential bias | 7 | Included in the last paragraph in Discussion section. |
| Interpretation | 20 | Give a cautious overall interpretation of results considering objectives, limitations, multiplicity of analyses, results from similar studies, and other relevant evidence | 7 | Included in the second last paragraph in Discussion section. |
| Generalisability | 21 | Discuss the generalisability (external validity) of the study results | 8 | Included in the conclusion section. |
| Other information | | |  |  |
| Funding | 22 | Give the source of funding and the role of the funders for the present study and, if applicable, for the original study on which the present article is based |  | Reported in the PeerJ submission system. |

*Give information separately for exposed and unexposed groups.

**Note:** An Explanation and Elaboration article discusses each checklist item and gives methodological background and published examples of transparent reporting. The STROBE checklist is best used in conjunction with this article (freely available on the Web sites of PLoS Medicine at http://www.plosmedicine.org/, Annals of Internal Medicine at http://www.annals.org/, and Epidemiology at http://www.epidem.com/). Information on the STROBE Initiative is available at www.strobe-statement.org.
